# Supplementary material for: Analysis of the Effects of Nutrient Intake and Dietary Habits on Depression in Korean Adults
Source: Nutrients. 2021 Apr 19;13(4):1360. doi: 10.3390/nu13041360 (PMC8073119; doi:10.3390/nu13041360)
Supplement: Supplementary file 1 [file nutrients-13-01360-s001.zip › nutrients-1146799-supplementary.pdf]

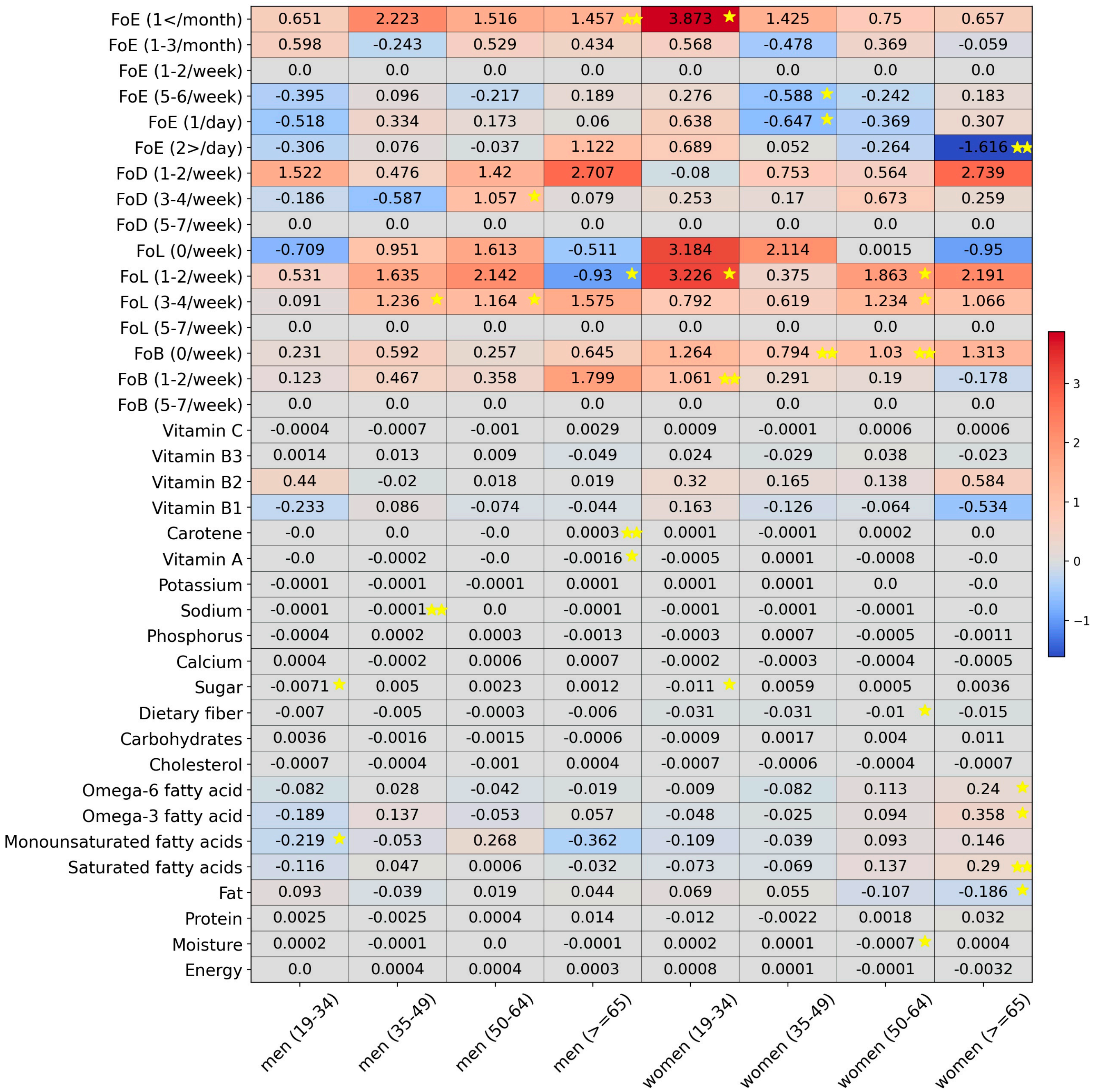

Figure S1. Heatmap of coefficients in the multiple regression models across the eight subgroups. The *p*-value significance was marked with yellow stars (* *p*-value < 0.05, ** *p*-value < 0.01). The coefficient values were also represented as text on the heatmap. FOE: Frequency of eating out, FOD: Frequency of dinner, FOL: Frequency of lunch, FOB: Frequency of breakfast
